# Supplementary material for: IL-10 Gene Rs1800871, Rs1800872, and Rs1800896 Polymorphisms and IL-10 Serum Levels Association with Pituitary Adenoma
Source: Biomedicines. 2022 Aug 8;10(8):1921. doi: 10.3390/biomedicines10081921 (PMC9405800; doi:10.3390/biomedicines10081921)
Supplement: Supplementary file 1 [file biomedicines-10-01921-s001.zip › biomedicines-1806681-supplementary.pdf]

biomedicines

MDPI

*Supplementary Materials*

# *IL-10* Gene Rs1800871, Rs1800872, and Rs1800896 Polymorphisms and IL-10 Serum Levels Association with Pituitary Adenoma

**Table S1.** Distributions of *IL – 10* rs1800871, rs1800872, rs1800896 genotypes and alleles in patients with PA and control groups.

| Gene | Genotype/allele | PA (n=106), *n* (%) | Control (n=192), *n* (%) | p-value | p-value HWE |
|---|---|---|---|---|---|
| ***IL – 10* (rs1800871)** | GG | 67 (63.2) | 103 (53.6) | 0.202 | 0.449 |
| | AG | 32 (30.2) | 78 (40,6) | | |
| | AA | 7 (6.6) | 11 (5,7) | | |
| | Viso: | 106 (100) | 192 (100) | | |
| | Allele:<br>G<br>A | <br>166 (78.3)<br>46 (21.7) | <br>284 (74.0)<br>100 (26.0) | 0.238 | |
| ***IL – 10* (rs1800872)** | GG | 68 (64.2) | 103 (53.6) | 0.148 | 0.449 |
| | TG | 31 (29.2) | 78 (40.6) | | |
| | TT | 7 (6.6) | 11 (5.7) | | |
| | Viso: | 106 (100) | 192 (100) | | |
| | Allele:<br>G<br>T | <br>167 (78.8)<br>45 (21.2) | <br>284 (74.0)<br>100 (26.0) | 0.190 | |
| ***IL – 10* (rs1800896)** | TT | 30 (28.3) | 49 (25.5) | 0.851 | 0.554 |
| | TC | 52 (49.1) | 100 (52.1) | | |
| | CC | 24 (22.6) | 43 (22.4) | | |
| | Viso: | 106 (100) | 192 (100) | | |
| | Allele:<br>T<br>C | <br>112 (52.8)<br>100 (47.2) | <br>198 (51.6)<br>186 (48.4) | 0.767 | |

**Table S2.** Binary logistic regression analysis of *IL – 10* rs1800871, rs1800872, rs1800896 in patients with PA and control groups.

***IL – 10* (rs1800871)**

| Model | Genotype/allele | OR (95% CI) | p - value | AIC |
|---|---|---|---|---|

| | | | | |
|---|---|---|---|---|
| Codominant | AG *vs*. GG<br>AA *vs*. GG | 0.631 (0.377 – 1.054)<br>0.978 (0.361 – 2.649) | 0.079<br>0.966 | 388.697 |
| Dominant | AG+AA *vs*. GG | 0.674 (0.414 – 1.095) | 0.111 | 389.849 |
| Recessive | AA *vs*. GG+AG | 1.163 (0.437 – 3.096) | 0.762 | 386.699 |
| Overdominant | AG *vs*. AA+GG | 0.632 (0.381 – 1.047) | 0.075 | 388.204 |
| Additive | G | 0.787 (0.528 – 1.173) | 0.239 | 388.531 |
| ***IL – 10*** **(rs1800872)** | | | | |
| Codominant | TG *vs*. GG<br>TT *vs*. GG | 0.602 (0.359 – 1.009)<br>0.964 (0.356 – 2.609) | 0.054<br>0.942 | 386.059 |
| Dominant | TG+TT *vs*. GG | 0.647 (0.397 – 1.053) | 0.080 | 389.849 |
| Recessive | TT *vs*. GG+TG | 1.163 (0.437 – 3.096) | 0.762 | 386.064 |
| Overdominant | TG *vs*. GG+TT | 0.604 (0.363 – 1.004) | 0.052 | 389.829 |
| Additive | G | 0.766 (0.513 – 1.144) | 0.192 | 386.059 |
| ***IL – 10*** **(rs1800896)** | | | | |
| Codominant | TC *vs*. TT<br>CC *vs*. TT | 1.177 (0.669 – 2.071)<br>1.073 (0.588 – 1.959) | 0.571<br>0.818 | 391.618 |
| Dominant | TC+CC *vs*. TT | 1.129 (0.702 – 1.814) | 0.617 | 389.938 |
| Recessive | CC *vs*. TT+TC | 1.014 (0.575 – 1.789) | 0.961 | 389.671 |
| Overdominant | TC *vs*. TT+CC | 1.152 (0.676 – 1.963) | 0.603 | 389.671 |
| Additive | T | 1.051 (0.785 – 1.408) | 0.738 | 389.618 |

OR: odds ratio; CI: confidence interval; AIC: Akaike information criterion; p-value: significance level (statistically significant when $p < 0.05$).

**Table S3.** Distributions of *IL – 10* rs1800871, rs1800872,rs1800896 genotypes and alleles in patients with PA and control groups by gender.

| Gene | Genotype | Males | | | Females | | |
|---|---|---|---|---|---|---|---|
| | | PA (n=46) *n*(%) | Control (n=63) *n* (%) | p - value | PA (n=60) *n* (%) | Control (n=129) *n* (%) | p - value |
| ***IL – 10* (rs1800871)** | AA | 3 (6.5) | 4 (6.3) | 0.393 | 4 (6.7) | 7 (5.4) | 0.361 |
| | AG | 16 (34.8) | 30 (47.6) | | 16 (26.7) | 48 (37.2) | |
| | GG | 27 (58.7) | 29 (46.0) | | 40 ( 66.7) | 74 (57.4) | |
| | Allele: | | | | | | |
| | A | 22 (23.9) | 38 (30.2) | 0.308 | 24 (20.0) | 62 (24.0) | 0.384 |
| | G | 70 (76.1) | 88 (69.8) | | 96 (80.0) | 196 (76.0) | |
| ***IL – 10* (rs1800872)** | GG | 28 (60.9) | 29 (46.0) | 0.276 | 40 (66.7) | 74 (57.4) | 0.361 |
| | TG | 15 (32.6) | 30 (47.6) | | 16 (26.7) | 48 (37.2) | |
| | TT | 3 (6.5) | 4 (6.3) | | 4 (6.7) | 7 (5.4) | |
| | Allele: | | | | | | |
| | G | 71 (77.2) | 88 (69.8) | 0.229 | 96 (80.0) | 196 (76.0) | 0.384 |
| | T | 21 (22.8) | 38 (30.2) | | 24 (20.0) | 62 (24.0) | |
| ***IL – 10* (rs1800896)** | CC | 12 (26.1) | 9 (14.3) | 0.261 | 12 (20.0) | 34 (26.4) | 0.306 |
| | TC | 24 (52.2) | 35 (55.6) | | 28 (46.7) | 65 (50.4) | |
| | TT | 10 (21.7) | 19 (30.2) | | 20 (33.3) | 30 (23.3) | |
| | Allele: | | | | | | |
| | C | 48 (52.2) | 53 (42.1) | 0.139 | 52 (43.3) | 133 (51.6) | 0.137 |
| | T | 44 (47.8) | 73 ( 57.9) | | 68 (56.7 ) | 125 (48.4) | |

**Table S4.** Binary logistic regression analysis of *IL – 10* rs1800871, rs1800872, rs1800896 in the PA and control groups by gender.

| Females | | | | |
|---|---|---|---|---|
| ***IL – 10* (rs1800871)** | | | | |
| **Model** | **Genotype/allele** | **OR (95% CI)** | **p - value** | **AIC** |
| Codominant | AG *vs.* GG | 1.463 (0.891 – 2.402) | 0.132 | 393.069 |
| | AA *vs.* GG | 1.295 (0.477 – 3.522) | 0.612 | |
| Dominant | AG+AA *vs.* GG | 1.439 (0.894 – 2.314) | 0.134 | 391.124 |
| Recessive | AA *vs.* GG+AG | 1.111 (0.417 – 2.954) | 0.834 | 393.330 |
| Overdominant | AG *vs.* AA+GG | 1.426 (0.878 – 2.316) | 0.151 | 391.322 |
| Additive | G | 1.290 (0.879 – 1.893) | 0.194 | 391.690 |

| | | | | |
|---|---|---|---|---|
| ***IL – 10* (rs1800872)** | | | | |
| Codominant | TG *vs*. GG<br>TT *vs*. GG | 0.786 (0.289 – 2.135)<br>1.105 (0.398 – 3.069) | 0.636<br>0.848 | 393.522 |
| Dominant | TG+TT *vs*. GG | 0.721 (0.448 – 1.160) | 0.178 | 391.559 |
| Recessive | TT *vs*. GG+TG | 1.111 (0.417 – 2.954) | 0.834 | 393.330 |
| Overdominant | TG *vs*. GG+TT | 1.373 (0.845 – 2.233) | 0.201 | 391.742 |
| Additive | G | 1.259 (0.858 – 1.847) | 0.240 | 391.997 |
| ***IL – 10* (rs1800896)** | | | | |
| Codominant | TC *vs*. TT<br>CC *vs*. TT | 0.914 (0.521 – 1.604)<br>0.720 (0.391 – 1.325) | 0.754<br>0.291 | 394.238 |
| Dominant | TC+CC *vs*. TT | 0.821 (0.512 – 1.317) | 0.413 | 392.703 |
| Recessive | CC *vs*. TT+TC | 0.742 (0.415 – 1.326) | 0.313 | 392.336 |
| Overdominant | TC *vs*. TT+CC | 1.008 (0.591 – 1.719) | 0.977 | 393.373 |
| Additive | T | 0.857 (0.638 – 1.151) | 0.305 | 392.309 |
| **Males** | | | | |
| ***IL – 10* (rs1800871)** | | | | |
| **Model** | **Genotype/allele** | **OR (95% CI)** | **p - value** | **AIC** |
| Codominant | AG *vs*. GG<br>AA *vs*. GG | 0.683 (0.416 – 1.122)<br>0.772 (0.284 – 2.099) | 0.683<br>0.772 | 393.069 |
| Dominant | AG+AA *vs*. GG | 0.695 (0.432 – 1.118) | 0.134 | 391.124 |
| Recessive | AA *vs*. GG+AG | 0.900 (0.339 – 2.395) | 0.834 | 393.330 |
| Overdominant | AG *vs*. AA+GG | 0.701 (0.432 – 1.139) | 0.151 | 391.322 |
| Additive | G | 0.775 (0.528 – 1.138) | 0.194 | 391.690 |
| ***IL – 10* (rs1800872)** | | | | |
| Codominant | TG *vs*. GG<br>TT *vs*. GG | 0.711 (0.433 – 1.168)<br>0.786 (0.289 – 2.135) | 0.178<br>0.636 | 393.522 |
| Dominant | TG+TT *vs*. GG | 0.721 (0.448 – 1.160) | 0.178 | 391.559 |
| Recessive | TT *vs*. GG+TG | 0.900 (0.339 – 2.395) | 0.834 | 393.330 |
| Overdominant | TG *vs*. GG+TT | 0.728 (0.448 – 1.184) | 0.201 | 391.742 |
| Additive | G | 0.794 (0.541 – 1.166) | 0.240 | 391.997 |
| ***IL – 10* (rs1800896)** | | | | |
| Codominant | TC *vs*. TT<br>CC *vs*. TT | 1.094 (0.624 – 1.919)<br>1.390 (0.755 – 2.559) | 0.754<br>0.291 | 394.238 |

| | | | | |
|---|---|---|---|---|
| Dominant | TC+CC *vs*. TT | 1.218 (0.759 – 1.954) | 0.413 | 392.703 |
| Recessive | CC *vs*. TT+TC | 1.348 (0.754 – 2.409) | 0.313 | 392.336 |
| Overdominant | TC *vs*. TT+CC | 0.992 (0.582 – 1.692) | 0.977 | 393.373 |
| Additive | T | 1.167 (0.869 – 1.569) | 0.305 | 392.309 |

OR: odds ratio; CI: confidence interval; AIC: Akaike information criteria; p-value: significance level (statistically significant when p < 0.05).

**Table S5.** Distributions of *IL – 10* rs1800871, rs1800872,rs1800896 genotypes and alleles in patients with PA and control groups by PA invasiveness.

| Gene | Genotype | Control (n=192) n (%) | Non-invasive PA (n=34) n (%) | p - value | Invasive PA (n=71) n (%) | p - value |
|---|---|---|---|---|---|---|
| ***IL – 10* (rs1800871)** | AA | 11 (5.70) | 1 (2.9) | 0.458 | 6 (8.5) | 0.233 |
| | AG | 78 (40.6) | 11 (32.4) | | 21 (29.6) | |
| | GG | 103 (53.6) | 22 (64.7) | | 44 (62.0) | |
| | Allele:<br>A<br>G | <br>100 (26.0)<br>284 (74.0) | <br>13 (19.1)<br>55 (80.9) | 0.224 | <br>33 (23.2)<br>109 (76.8) | 0.512 |
| ***IL – 10* (rs1800872)** | GG | 103 (53.6) | 22 (64.7) | 0.458 | 45 (63.4) | 0.163 |
| | TG | 78 (40.6) | 11 (32.4) | | 20 (28.2) | |
| | TT | 11 (5.7) | 1 (2.9) | | 6 (8.5) | |
| | Allele:<br>G<br>T | <br>284 (74.0)<br>100 (26.0) | <br>55 (80.9)<br>13 (19.1) | 0.224 | <br>110 (77.5)<br>32 (22.5) | 0.410 |
| ***IL – 10* (rs1800896)** | CC | 43 (22.4) | 4 (11.8) | 0.371 | 20 (28.2) | 0.450 |
| | TC | 100 (52.1) | 20 (58.8) | | 31 (43.7) | |
| | TT | 49 (25.5) | 10 (29.4) | | 20 (28.2) | |
| | Allele:<br>C<br>T | <br>186 (48.4)<br>198 (51.6) | <br>28 (41.2)<br>40 (58.8) | 0.269 | <br>71 (50.0)<br>71 (50.0) | 0.750 |

**Table S6.** Binary logistic regression analysis of *IL – 10* rs1800871, rs1800872, rs1800896 in the PA and control groups by PA invasiveness.

| ***IL – 10* (rs1800871)** | | | | |
|---|---|---|---|---|
| **Model** | **Genotype/allele** | **OR (95% CI)** | **p - value** | **AIC** |
| **Non-invasive PA** | | | | |
| Codominant | AG *vs*. GG<br>AA *vs*. GG | 1.515 (0.693 – 3.308)<br>2.350 (0.288 - 19.153) | 0.298<br>0.425 | 193.779 |

| | | | | |
|---|---|---|---|---|
| Dominant | AG+AA *vs.* GG | 1.584 (0.742 – 3.382) | 0.234 | 191.958 |
| Recessive | AA *vs.* GG+AG | 2.006 (0.250 - 16.061) | 0.512 | 192.896 |
| Overdominant | AG *vs.* AA+GG | 1.431 (0.660 – 3.102) | 0.365 | 192.566 |
| Additive | G | 1.521 (0.784 – 2.951) | 0.215 | 191.780 |
| **Invasive PA** | | | | |
| Codominant | AG *vs.* GG<br>AA *vs.* GG | 1.587 (0.873 – 2.883)<br>0.783 (0.273 – 2.250) | 0.130<br>0.650 | 307.816 |
| Dominant | AG+AA *vs.* GG | 1.408 (0.807 – 2.457) | 0.228 | 307.305 |
| Recessive | AA *vs.* GG+AG | 0.658 (0.234 – 1.852) | 0.428 | 308.171 |
| Overdominant | AG *vs.* AA+GG | 1.629 (0.907 – 2.925) | 0.102 | 304.018 |
| Additive | G | 1.163 (0.741 – 1.825) | 0.513 | 305.816 |
| ***IL – 10* (rs1800872)** | | | | |
| **Non-invasive PA** | | | | |
| Codominant | TG *vs.* GG<br>TT *vs.* GG | 1.515 (0.693 – 3.308)<br>2.350 (0.288 - 19.153) | 0.298<br>0.425 | 193.779 |
| Dominant | TG+TT *vs.* GG | 1.584 (0.742 – 3.382) | 0.234 | 191.958 |
| Recessive | TT *vs.* GG+TG | 2.006 (0.250 - 16.061) | 0.512 | 192.896 |
| Overdominant | TG *vs.* GG+TT | 1.431 (0.660 – 3.102) | 0.365 | 192.566 |
| Additive | G | 1.521 (0.784 – 2.951) | 0.215 | 191.780 |
| **Invasive PA** | | | | |
| Codominant | TG *vs.* GG<br>TT *vs.* GG | 1.704 (0.932 – 3.115)<br>0.801 (0.279 – 2.299) | 0.083<br>0.680 | 307.073 |
| Dominant | TG+TT *vs.* GG | 1.496 (0.854 – 2.618) | 0.159 | 306.757 |
| Recessive | TT *vs.* GG+TG | 0.658 (0.234 – 1.852) | 0.428 | 308.171 |
| Overdominant | TG *vs.* GG+TT | 1.745 (0.965 – 3.153) | 0.065 | 305.240 |
| Additive | G | 1.209 (0.768 – 1.903) | 0.413 | 308.091 |
| ***IL – 10* (rs1800896)** | | | | |
| **Non-invasive PA** | | | | |
| Codominant | TC *vs.* TT<br>CC *vs.* TT | 0.980 (0.426 – 2.253)<br>2.150 (0.694 – 6.665) | 0.962<br>0.185 | 193.194 |
| Dominant | TC+CC *vs.* TT | 1.314 (0.627 – 2.753) | 0.469 | 192.881 |
| Recessive | CC *vs.* TT+TC | 2.164 (0.723 – 6.483) | 0.168 | 191.197 |
| Overdominant | TC *vs.* TT+CC | 0.822 (0.367 – 1.841) | 0.634 | 193.189 |

| | | | | |
|---|---|---|---|---|
| Additive | T | 1.338 (0.820 – 2.185) | 0.244 | 191.983 |
| **Invasive PA** | | | | |
| Codominant | TC *vs.* TT<br>CC *vs.* TT | 0.760 (0.393 – 1.466)<br>0.667 (0.342 – 1.297) | 0.412<br>0.233 | 309.182 |
| Dominant | TC+CC *vs.* TT | 0.713 (0.412 – 1.233) | 0.226 | 307.301 |
| Recessive | CC *vs.* TT+TC | 0.736 (0.396 – 1.366) | 0.331 | 307.847 |
| Overdominant | TC *vs.* TT+CC | 0.874 (0.475 – 1.609) | 0.665 | 308.588 |
| Additive | T | 0.812 (0.585 – 1.127) | 0.213 | 307.234 |

OR: odds ratio; CI: confidence interval; AIC: Akaike information criteria; p-value: significance level (statistically significant when $p < 0.05$).

**Table S7.** Distributions of *IL – 10* rs1800871, rs1800872, rs1800896 genotypes and alleles in patients with PA and control groups by PA recurrence.

| Gene | Genotype | Control (n=192), *n* (%) | Without recurrence (n=81), *n* (%) | p - value | With recurrence (n=21), *n* (%) | p - value |
|---|---|---|---|---|---|---|
| ***IL – 10* (rs1800871)** | AA | 11 (5.7) | 6 (7.4) | 0.226 | 1 (4.8) | 0.771 |
| | AG | 78 (40.6) | 24 (29.6) | | 7 (33.3) | |
| | GG | 103 (53.6) | 51 (63.0) | | 13 (61.9) | |
| | Allele:<br>A<br>G | <br>100 (26.0)<br>284 (74.0) | <br>36 (22.2)<br>126 (77.8) | 0.346 | <br>9 (21.4)<br>33 (78.6) | 0.515 |
| ***IL – 10* (rs1800872)** | GG | 103 (53.6) | 52 (64.2) | 0.159 | 13 (61.9) | 0.771 |
| | TG | 78 (40.6) | 23 (28.4) | | 7 (33.3) | |
| | TT | 11 (5.7) | 6 (7.4) | | 1 (4.8) | |
| | Allele:<br>G<br>T | <br>284 (74.0)<br>100 (26.0) | <br>127 (78.4)<br>35 (21.6) | 0.272 | <br>33 (78.6)<br>9 (21.4) | 0.515 |
| ***IL – 10* (rs1800896)** | CC | 43 (22.4) | 18 (22.2) | 0.878 | 5 (23.8) | 0.924 |
| | TC | 100 (52.1) | 40 (49.4) | | 10 (47.6) | |
| | TT | 49 (25.5) | 23 (28.4) | | 6 (28.6) | |
| | Allele:<br>C<br>T | <br>186 (48.4)<br>198 (51.6) | <br>76 (46.9)<br>86 (53.1) | 0.745 | <br>20 (47.6)<br>22 (52.4) | 0.920 |

**Table S8.** Binary logistic regression analysis of *IL – 10* rs1800871, rs1800872, rs1800896 in the PA and control groups by PA recurrence.

| Model | Genotype/allele | OR (95% CI) | p - value | AIC |
|---|---|---|---|---|
| ***IL – 10* (rs1800871)** | | | | |
| **Without recurrence** | | | | |
| Codominant | AG *vs.* GG<br>AA *vs.* GG | 1.609 (0.912 – 2.838)<br>0.908 (0.318 – 2.594) | 0.100<br>0.857 | 332.957 |
| Dominant | AG+AA *vs.* GG | 1.496 (0.862 – 2.503) | 0.157 | 331.963 |
| Recessive | AA *vs.* GG+AG | 0.760 (0.271 – 2.129) | 0.601 | 333.726 |
| Overdominant | AG *vs.* AA+GG | 1.625 (0.931 – 2.837) | 0.088 | 330.989 |
| Additive | G | 1.232 (0.797 – 1.905) | 0.347 | 333.091 |
| **With recurrence** | | | | |
| Codominant | AG *vs.* GG<br>AA *vs.* GG | 1.406 (0.536 - 3.691)<br>1.388 (0.166 - 11.646) | 0.488<br>0.762 | 140.636 |
| Dominant | AG+AA *vs.* GG | 1.404 (0.557 - 3.542) | 0.472 | 138.636 |
| Recessive | AA *vs.* GG+AG | 1.215 (0.149 - 9.912) | 0.855 | 139.127 |
| Overdominant | AG *vs.* AA+GG | 1.368 (0.528 - 3.545) | 0.518 | 138.734 |
| Additive | G | 1.307 (0.594 - 2.879) | 0.506 | 138.703 |
| ***IL – 10* (rs1800872)** | | | | |
| **Without recurrence** | | | | |
| Codominant | TG *vs.* GG<br>TT *vs.* GG | 1.712 (0.966 – 3.034)<br>0.926 (0.324 – 2.643) | 0.066<br>0.885 | 332.228 |
| Dominant | TG+TT vs. GG | 1.549 (0.907 – 2.647) | 0.109 | 331.379 |
| Recessive | TT *vs.* GG+TG | 0.760 (0.271 – 2.129) | 0.601 | 333.726 |
| Overdominant | TG *vs.* GG+TT | 1.725 (0.983 – 3.028) | 0.057 | 331.248 |
| Additive | G | 1.276 (0.824 – 1.978) | 0.275 | 332.772 |
| **With recurrence** | | | | |
| Codominant | TG *vs.* GG<br>TT *vs.* GG | 1.406 (0.536 – 3.691)<br>1.388 (0.166 - 11.646) | 0.488<br>0.762 | 140.636 |
| Dominant | TG+TT *vs.* GG | 1.404 (0.557 – 3.542) | 0.472 | 138.636 |
| Recessive | TT *vs.* GG+TG | 1.215 (0.149 – 9.912) | 0.855 | 139.127 |
| Overdominant | TG *vs.* GG+TT | 1.368 (0.528 – 3.545) | 0.518 | 138.734 |
| Additive | G | 1.307 (0.594 – 2.879) | 0.506 | 138.703 |

| | | | | |
|---|---|---|---|---|
| ***IL – 10*** **(rs1800896)** | | | | |
| **Without recurrence** | | | | |
| Codominant | TC *vs.* TT<br>CC *vs.* TT | 0.852 (0.460 – 1.578)<br>0.956 (0.493 – 1.851) | 0.611<br>0.893 | 335.734 |
| Dominant | TC+CC *vs.* TT | 0.898 (0.534 – 1.509) | 0.683 | 333.826 |
| Recessive | CC *vs.* TT+TC | 1.010 (0.541 – 1.885) | 0.975 | 333.992 |
| Overdominant | TC *vs.* TT+CC | 0.864 (0.483 – 1.546) | 0.623 | 333.752 |
| Additive | T | 0.962 (0.698 – 1.326) | 0.813 | 333.937 |
| **With recurrence** | | | | |
| Codominant | TC *vs.* TT<br>CC *vs.* TT | 0.817 (0.281 – 2.377)<br>0.860 (0.270 – 2.666) | 0.710<br>0.794 | 141.005 |
| Dominant | TC+CC *vs.* TT | 0.836 (0.339 – 2.061) | 0.698 | 139.011 |
| Recessive | CC *vs.* TT+TC | 0.923 (0.320 – 2.665) | 0.883 | 139.141 |
| Overdominant | TC *vs.* TT+CC | 0.857 (0.315 – 2.330) | 0.762 | 139.072 |
| Additive | T | 0.916 (0.530 – 1.583) | 0.753 | 139.064 |

OR: odds ratio; CI: confidence interval; AIC: Akaike information criteria; p-value: significance level (statistically significant when $p < 0.05$).

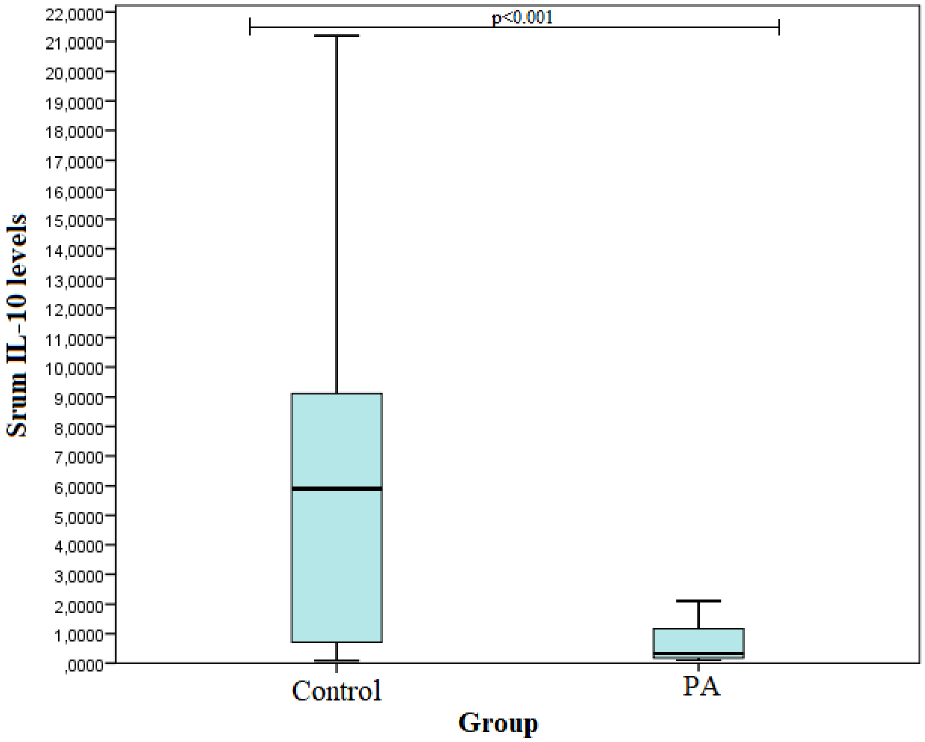


**Figure S1.** Serum IL-10 levels in PA and control groups.

**Table S9.** Associations between *IL-10* haplotypes and risk of pituitary adenoma.

| Haplotype | rs1800871 | rs1800872 | rs1800896 | Frequency (%) | | | OR (95% CI) | p-value |
|---|---|---|---|---|---|---|---|---|
| | | | | PA | Controls | Total | | |
| 1 | G | G | C | 47.17 | 48.08 | 47.75 | 1.00 | --- |
| 2 | G | G | T | 31.13 | 25.88 | 27.75 | 1.24 (0.83 – 1.86) | 0.29 |
| 3 | A | T | T | 21.23 | 25.68 | 24.10 | 0.84 (0.54 – 1.29) | 0.42 |
| rare | * | * | * | NA | NA | 0.40 | 2.02 (0.12 – 33.38) | 0.62 |

OR – odds ratio; CI – confident interval; p – significance level when p=0.05; AIC – Akaike information criteria; NA – not applicable; rare – pooled haplotypes with frequencies <1%.
